# Nutrigenomics and Epigenetic Regulation in Cancer Prevention: Molecular Mechanisms, Dietary Bioactives, and Translational Perspectives

**DOI:** 10.3390/nu18183101

**Published:** 2026-09-21

**Authors:** Grigoris Risvas, Stavros P. Derdas

**Affiliations:** Department of Dietetics, School of Health, Aegean College, 10564 Athens, Greece

**Keywords:** nutrigenomics, nutritional epigenetics, cancer prevention, DNA methylation, histone modification, microRNAs, diet–gene interaction

## Abstract

Cancer development is influenced not only by genetic alterations but also by epigenetic dysregulation driven by environmental and lifestyle factors, particularly diet. Nutrigenomics investigates how dietary components interact with the genome to influence gene expression, while nutritional epigenetics focuses on diet-induced modifications such as DNA methylation, histone modifications and non-coding RNA regulation. Increasing evidence indicates that bioactive dietary compounds can modulate epigenetic mechanisms involved in carcinogenesis, highlighting their potential role in cancer prevention. This narrative review synthesizes recent evidence, with emphasis on literature published between 2015 and 2025 and an updated search through September 2026 on the molecular interplay between diet, epigenetic regulation and cancer risk. We examine how key dietary factors—including methyl donors involved in one-carbon metabolism, polyphenols, short-chain fatty acids and omega-3 fatty acids—modulate epigenetic regulators such as DNA methyltransferases and histone deacetylases. Particular emphasis is placed on the emerging Nutrigenomics–Microbiome–Epigenome Axis, whereby microbiota-derived metabolites influence host chromatin architecture and gene expression pathways relevant to tumor suppression and inflammation. In addition, we discuss current evidence linking dietary patterns, including Mediterranean and Western diets, with distinct epigenomic signatures associated with cancer susceptibility. The review further explores the potential of epigenetic biomarkers and epigenome-wide association studies to support precision nutrition strategies for cancer prevention. Collectively, accumulating evidence suggests that diet-driven epigenetic plasticity may contribute to cancer prevention. However, the human evidence remains heterogeneous, and large, well-designed intervention studies with standardized epigenetic endpoints are required before mechanistic findings can be translated into clinically actionable precision nutrition strategies.

## 1. Introduction

Cancer risk reflects the interaction of inherited susceptibility with modifiable exposures that can alter cellular regulatory programs over time. Among these exposures, diet is particularly relevant because it influences metabolic signaling, inflammatory tone, microbial metabolism, and the availability of substrates and cofactors required for epigenetic regulation [1]. Updated GLOBOCAN estimates reported approximately 20 million new cancer cases and 9.7 million cancer deaths worldwide in 2022, emphasizing the continuing need for biologically grounded prevention strategies [2].

Beyond irreversible genetic mutations, cancer development is critically shaped by epigenetic dysregulation. Epigenetic mechanisms—including DNA methylation, histone modifications, and non-coding RNA regulation—govern gene expression without altering the underlying DNA sequence and are highly dynamic and environmentally responsive [3,4]. These modifications are potentially reversible and cooperate with genetic alterations to promote tumor initiation, progression and metastasis [3,4]. Environmental exposures, including dietary factors, can induce persistent epigenetic changes that influence pathways central to carcinogenesis, such as inflammation, oxidative stress, DNA repair, and cell cycle regulation [5,6].

Nutrigenomics and nutritional epigenetics provide complementary frameworks for examining how dietary exposures influence gene regulation. Nutrigenomics addresses genome-wide responses to nutrients and dietary patterns, whereas nutritional epigenetics focuses on diet-responsive DNA methylation, chromatin states and regulatory RNA networks [7,8,9,10]. High-throughput epigenomic and multi-omics technologies have expanded this field from candidate-gene observations toward systems-level analyses, but translation remains constrained by tissue specificity, exposure measurement error, interindividual variability and limited prospective human validation [11].

Accumulating evidence demonstrates that bioactive dietary compounds—including methyl donors involved in one-carbon metabolism, polyphenols, short-chain fatty acids (SCFAs) and omega-3 fatty acids—can modulate epigenetic enzymes such as DNA methyltransferases (DNMTs) and histone deacetylases (HDACs) [5,12,13,14]. In parallel, diet-induced alterations in gut microbiota composition influence host epigenetic landscapes through microbial-derived metabolites, particularly butyrate, thereby establishing a Nutrigenomics–Microbiome–Epigenome Axis relevant to cancer prevention [12,15,16,17,18].

**Figure 1:**
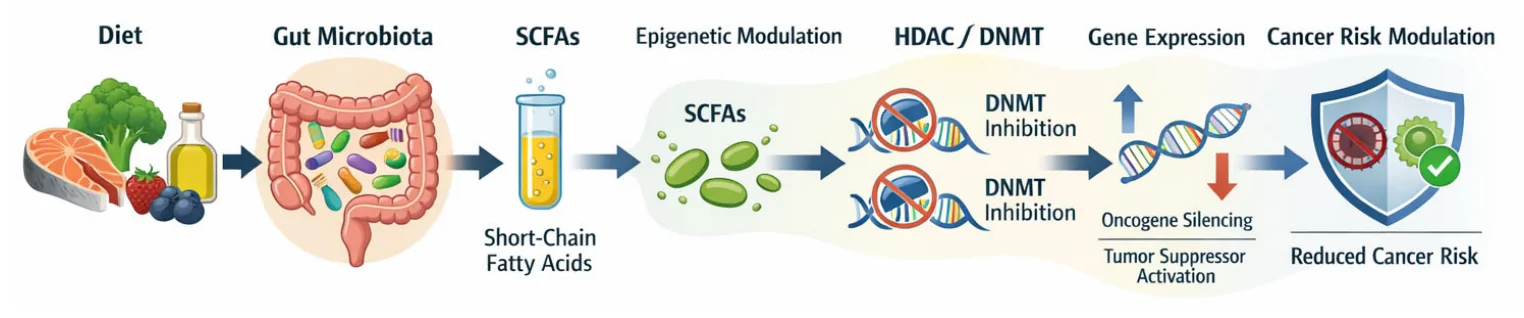
Nutrigenomics–Microbiome–Epigenome Axis in cancer risk modulation.

The principal knowledge gap is therefore not whether dietary exposures can influence epigenetic regulation, but which diet-responsive molecular changes are reproducible in humans, causally related to cancer-relevant pathways, and sufficiently robust to support risk stratification or personalized intervention. Previous reviews have addressed nutritional epigenetics, nutrigenomics or diet-related mechanisms separately [4,7,10]. The present review extends this literature by critically integrating dietary bioactives and patterns, microbial metabolism, contemporary epigenetic mechanisms, human biomarker evidence, and translational barriers within a unified Nutrigenomics–Microbiome–Epigenome Axis. Figure 1 introduces the core diet–microbiome–epigenome component of this framework, which is subsequently expanded in the human and translational sections.

## 2. Methods: Review Design and Literature Search

This article is a narrative review, rather than a systematic or scoping review. Its purpose is to provide a critical mechanistic and translational synthesis of evidence linking diet, epigenetic regulation, the gut microbiome, and cancer prevention. A structured literature search was used to improve transparency and coverage, but the review was not prospectively registered and was not designed to provide an exhaustive systematic inventory of all eligible studies. PubMed/MEDLINE was used as the principal bibliographic database, supplemented by backward citation tracking of relevant reviews and primary studies. The core search covered January 2015 through December 2025; seminal earlier studies were retained when required to establish foundational mechanisms. The search was updated through 2 September 2026 for the revision.

Search concepts were combined with Boolean operators. A representative PubMed/MEDLINE search string was: (nutrigenomics OR “nutritional epigenetics” OR diet OR dietary OR nutrition) AND (cancer OR neoplas* OR carcinogenesis) AND (“DNA methylation” OR DNMT OR TET OR “histone modification” OR HDAC OR HAT OR “chromatin accessibility” OR ATAC-seq OR microRNA OR miRNA OR epigenom*) AND (polyphenol OR curcumin OR resveratrol OR EGCG OR sulforaphane OR folate OR “short-chain fatty acid” OR microbiome OR “precision nutrition”). Targeted searches were additionally conducted for Mediterranean and Western dietary patterns, epigenome-wide association studies (EWAS), epigenetic clocks, LINE-1 methylation, and human intervention studies. Reference lists of eligible papers were examined to identify additional relevant publications.

Study selection was conducted in two stages: titles and abstracts were first assessed for relevance to diet-responsive epigenetic regulation and cancer prevention, followed by full-text assessment of potentially relevant reports. Inclusion prioritized randomized or controlled human interventions, prospective and population-based studies, systematic reviews and meta-analyses, EWAS and other human omics studies, together with mechanistic studies directly informing biological plausibility. Preclinical studies were included when they addressed mechanisms for which corresponding human evidence was limited or unavailable. Reports were excluded if they lacked direct relevance to nutrition–epigenome interactions, provided insufficient methodological detail, were conference abstracts without full data, or duplicated evidence available in a more complete publication. Because the objective was interpretive synthesis rather than exhaustive systematic evidence mapping, no PRISMA flow diagram, formal risk-of-bias instrument, or quantitative meta-analysis was applied. Evidence was assessed qualitatively according to study design, biological plausibility, consistency, reproducibility, exposure relevance, and translational applicability.

Accordingly, the search strategy should be interpreted as structured and reproducible at the level of the principal database, search concepts, and eligibility framework, while recognizing the inherent limitations of a narrative review: study identification and weighting were guided by relevance to the review questions rather than by a protocol-defined exhaustive selection process. This distinction is maintained throughout the manuscript when interpreting the strength of human versus preclinical evidence.

## 3. Epigenetic Mechanisms in Carcinogenesis

Epigenetic regulation encompasses heritable and reversible changes in gene expression that do not involve alterations in DNA sequence. The principal epigenetic mechanisms include DNA methylation, histone modifications, and regulation by non-coding RNAs, all of which are frequently dysregulated in cancer [3,4].

### 3.1. DNA Methylation

DNA methylation involves the transfer of a methyl group to the 5′ position of cytosine residues, predominantly within CpG dinucleotides, and is catalyzed by DNA methyltransferases (DNMT1, DNMT3A, and DNMT3B) [14]. In normal cells, appropriately regulated DNA methylation contributes to genomic stability, genomic imprinting, cellular identity, and transcriptional regulation [14,19].

Cancer cells exhibit profound alterations in DNA methylation patterns, characterized by global hypomethylation and site-specific hypermethylation of CpG islands in promoter regions of tumor suppressor genes [3,14]. These aberrant methylation patterns contribute to genomic instability, oncogene activation, and silencing of genes involved in cell cycle control, apoptosis, and DNA repair [6].

Dietary factors can influence DNA methylation through one-carbon metabolism, which supplies S-adenosylmethionine (SAM), the universal methyl donor for DNA and histone methylation [13,14,20]. Perturbation of folate- and methionine-cycle substrates or cofactors can alter SAM/SAH balance and methylation capacity; associations with cancer susceptibility are context-, tissue- and exposure-dependent rather than uniformly causal [13,14,20].

### 3.2. Histone Modifications

Histones are core chromatin proteins that undergo post-translational modifications such as acetylation, methylation, phosphorylation, and ubiquitination, which collectively regulate chromatin structure and gene accessibility [4]. Histone acetylation, mediated by histone acetyltransferases (HATs), generally promotes transcription, whereas histone deacetylases (HDACs) repress gene expression by condensing chromatin [12,17].

Aberrant expression and activity of histone-modifying enzymes are commonly observed in cancer and contribute to oncogenic transcriptional programs [3]. Importantly, several dietary bioactive compounds have been shown to modulate histone acetylation and methylation by inhibiting HDACs or influencing histone methyltransferases, thereby restoring the expression of tumor suppressor genes [5,21].

### 3.3. Non-Coding RNAs

Non-coding RNAs, particularly microRNAs (miRNAs) and long non-coding RNAs (lncRNAs), represent an additional epigenetic regulatory layer that controls gene expression post-transcriptionally [6]. Dysregulated miRNA expression is a hallmark of many cancers and affects key signaling pathways involved in proliferation, invasion, and apoptosis [3].

Emerging evidence indicates that dietary components, including polyphenols and fatty acids, can modulate miRNA expression profiles, thereby indirectly influencing epigenetic networks relevant to cancer prevention [5,7].

### 3.4. TET Enzymes, Histone Acetyltransferases, and Chromatin Accessibility

Contemporary epigenetic regulation extends beyond DNMT- and HDAC-mediated processes. Ten-eleven translocation (TET) dioxygenases catalyze the stepwise oxidation of 5-methylcytosine and participate in active DNA demethylation, enhancer regulation, and cell-state transitions; dysregulation of TET activity is increasingly implicated in inflammation and cancer [19]. Histone acetyltransferases (HATs) act as epigenetic “writers” that deposit acetyl groups on histones and other proteins, thereby influencing chromatin accessibility and transcriptional competence. Their effects are context-dependent, and altered HAT activity can support either tumor-suppressive or oncogenic programs [22].

These regulatory processes are increasingly interrogated using epigenome-wide technologies. Assay for Transposase-Accessible Chromatin using sequencing (ATAC-seq), together with ChIP-seq, DNA methylation profiling, and single-cell multi-omics, enables genome-wide mapping of accessible chromatin and regulatory elements. Such approaches are important for distinguishing broad biochemical effects of dietary compounds from locus-specific regulatory changes that are reproducible in relevant human tissues [23].

## 4. Nutrigenomics and Epigenetic Plasticity

Nutrigenomics investigates how nutrients and dietary patterns influence gene expression and cellular function. Epigenetic plasticity enables cells to dynamically respond to nutritional cues, particularly during critical periods such as early development and aging [24].

Interindividual variability in nutrigenomic responses is influenced by genetic polymorphisms, baseline epigenetic status, age, sex, and gut microbiota composition [7,10]. This heterogeneity complicates interpretation and reproducibility of nutritional intervention studies and is one rationale for investigating stratified or personalized nutrition approaches, although clinical benefit remains to be demonstrated [7,10,25].

## 5. Dietary Components Modulating Epigenetic Mechanisms

### 5.1. Methyl Donors and One-Carbon Metabolism

One-carbon metabolism integrates dietary folate, vitamins B12 and B6, methionine, and choline to generate SAM for DNA and histone methylation reactions [13,14]. Folate deficiency has been associated with global DNA hypomethylation, chromosomal instability, and increased risk of colorectal and other cancers [13,14].

However, excessive folate intake may promote the progression of pre-neoplastic lesions, emphasizing the importance of dose, timing, and individual risk profile in dietary recommendations [14].

### 5.2. Polyphenols and Phytochemicals

Dietary polyphenols, including curcumin, resveratrol, and epigallocatechin-3-gallate (EGCG), have demonstrated epigenetic activity in experimental systems [5,21]. Sulforaphane is considered separately because it is not a polyphenol; chemically, it is an aliphatic isothiocyanate generated from the glucosinolate glucoraphanin by myrosinase. Reported mechanisms for these compounds include modulation of DNMTs, HDACs, HATs, and regulatory RNAs. Importantly, food-level exposure is often substantially lower than the concentrations used in cell culture or pharmacological supplementation studies, and intestinal metabolism further changes the circulating molecular species. Consequently, mechanistic plausibility should not be interpreted as evidence of preventive efficacy at habitual dietary intakes [26,27,28,29,30]. The principal dietary components, their proposed epigenetic targets and mechanisms, associated cancer models, evidence levels, and key references are summarized in Table 1.

In addition to direct epigenetic modulation, polyphenols exert antioxidant and anti-inflammatory effects, reinforcing their multifaceted role in cancer prevention [5,31].

Sulforaphane is an aliphatic isothiocyanate derived from glucoraphanin in broccoli and other cruciferous vegetables. Its delivered dose depends strongly on glucoraphanin content, plant myrosinase activity, food processing, and the gut microbiota. In a human crossover study, 40 g of fresh broccoli sprouts provided approximately 150 μmol glucoraphanin, while intervention preparations used in other studies have delivered 100–200 μmol sulforaphane equivalents; these exposures illustrate that standardized experimental doses may exceed those obtained from ordinary servings of mature cruciferous vegetables [30].

**Table 1:** Summary of dietary components and dietary patterns that modulate epigenetic mechanisms relevant to cancer prevention, including their principal molecular targets, associated cancer models, evidence levels, and key references.

Dietary Component	Principal Epigenetic Target/Mechanism	Cancer Model(s)	Evidence Level	Key References
Folate (methyl donor)	DNA methylation via one-carbon metabolism (SAM synthesis); DNMT activity modulation	Colorectal, other solid tumors	Human observational studies; intervention trials	[13,14]
Curcumin	DNMT inhibition; HDAC modulation; miRNA regulation	Colorectal, breast	Preclinical; human pharmacokinetic/biomarker studies	[5,32]
Resveratrol	Histone modification; SIRT1 activation; miRNA modulation	Breast, prostate	Preclinical; early-phase human biomarker study	[5,33,34]
EGCG (green tea polyphenol)	DNMT inhibition; histone acetylation modulation	Prostate, colorectal	Preclinical; human tissue-exposure studies	[5,26,27]
Sulforaphane (isothiocyanate)	HDAC inhibition; chromatin remodeling	Colorectal	Preclinical; human bioavailability studies	[5,21,30]
Butyrate (SCFA)	HDAC inhibition; histone hyperacetylation	Colorectal cancer	Mechanistic; physiologic human exposure data	[12,17,35,36]
Omega-3 fatty acids	miRNA modulation; anti-inflammatory epigenetic signaling	Breast, colorectal	Observational; mechanistic	[1,5]
Mediterranean diet (pattern)	Favorable DNA methylation signatures; inflammation-related gene modulation	Multiple cancers	Population studies	[31,37,38]
Western diet (pattern)	Global DNA hypomethylation; oncogene activation; pro-inflammatory epigenetic profile	Colorectal, breast	Epidemiological; animal	[3,39,40]
Caloric restriction/fasting	SIRT1 activation; AMPK–mTOR axis; histone acetylation changes	Multiple tumor models	Preclinical; translational	[21,33,41]

**Figure 2:**
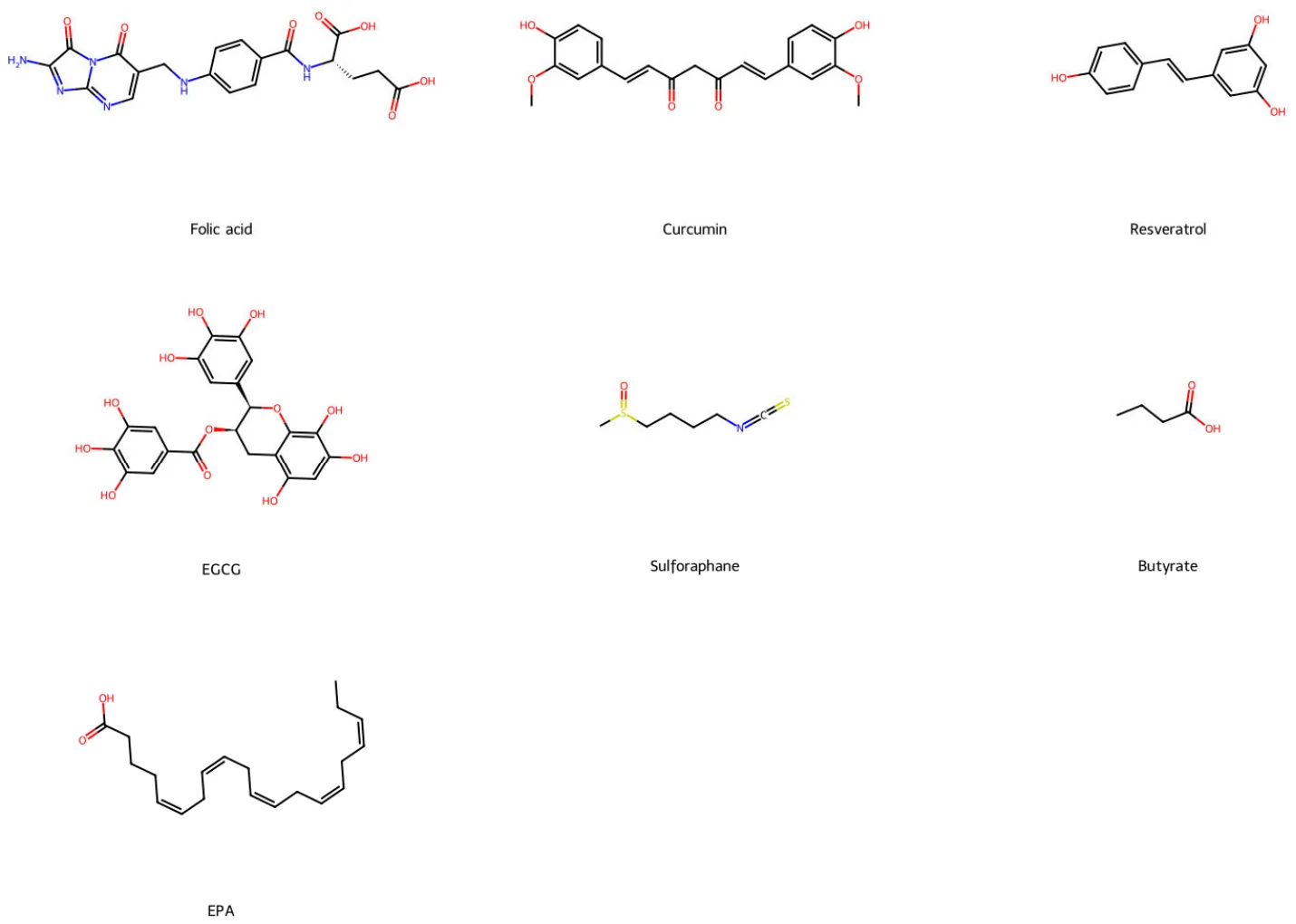
Chemical structures of representative pure compounds discussed in the manuscript. EPA is shown as a representative long-chain omega-3 polyunsaturated fatty acid. Sulforaphane is shown separately from polyphenols to emphasize its classification as an aliphatic isothiocyanate.

A key translational issue is the difference between concentrations used in mechanistic experiments and those achievable through ordinary foods. Table 2 therefore provides representative food-level exposure ranges for the principal compounds discussed in this review. These values should not be treated as fixed composition data: cultivar, storage, cooking, brewing, serving size, enzymatic conversion, and host metabolism can shift actual exposure by several-fold. Figure 2 provides the chemical structures of representative pure compounds to clarify their distinct chemical classes.

**Table 2:** Approximate dietary exposure to representative bioactive compounds discussed in this review. Values are illustrative rather than universal because cultivar, food processing, preparation, serving size, and analytical method can substantially alter exposure. DFE, dietary folate equivalents.

Bioactive/Nutrient	Representative Dietary Source	Approximate Food-Level Exposure or Concentration	Translational Relevance
Folate	Boiled spinach; legumes	~131 μg DFE per 1/2 cup boiled spinach; ~105 μg DFE per 1/2 cup boiled black-eyed peas [28]	Habitual intake is in μg/day, unlike pharmacological folic acid dosing; effects depend on baseline status and one-carbon metabolism.
Curcumin	Turmeric (Curcuma longa)	Curcuminoids comprise ~1–6% of dry turmeric; curcumin is ~60–70% of the curcuminoid fraction. A 2 g culinary portion therefore generally provides only tens of milligrams of curcumin [29].	Many human trials use 2–4 g/day purified curcumin, far above normal culinary exposure, and systemic bioavailability is low.
Resveratrol	Grapes and red wine	Greek red wines contained ~0.35–1.99 mg/L trans-resveratrol; a 150 mL serving therefore provides roughly 0.05–0.30 mg in that dataset [42].	Experimental supplemental doses are usually orders of magnitude above dietary exposure; wine is not advocated as a preventive delivery vehicle.
EGCG	Brewed green tea	Measured green tea infusions have contained ~117–442 mg/L EGCG (about 28–106 mg per 240 mL cup), depending strongly on tea and brewing conditions [27].	Clinical extracts often deliver several hundred mg EGCG/day; beverage exposure is variable and lower than many intervention doses.
Sulforaphane/glucoraphanin	Broccoli sprouts and cruciferous vegetables	In one crossover study, 40 g fresh broccoli sprouts provided ~150 μmol glucoraphanin; conversion to sulforaphane depends on myrosinase activity [30].	Food processing and microbiota cause large interindividual variation in systemic sulforaphane exposure.
Butyrate (SCFA)	Microbial fermentation of fiber/resistant starch	Not a meaningful dietary intake as purified butyrate. Total colonic SCFAs are ~80–130 mmol/kg; butyrate is commonly ~20% of the major SCFA pool [36].	Luminal exposure can reach millimolar levels, whereas systemic concentrations are far lower; site of action is therefore critical.
EPA/DHA (omega-3 PUFAs)	Oily fish	A cooked 85 g (3 oz) serving of Atlantic salmon provides approximately 0.35–0.59 g EPA and 1.22–1.24 g DHA [43].	Dietary exposure can reach gram-scale amounts, but epigenetic effects remain dependent on dose, tissue, and background diet.

## 6. Gut Microbiota, Diet, and Epigenetic Regulation in Cancer Prevention

The gut microbiota is increasingly recognized as a mediator linking dietary exposures with host metabolism, immune signaling, and epigenetic regulation [15,16,44,45]. Dietary components influence microbial composition and metabolic activity, generating metabolites capable of modifying chromatin-associated processes and gene expression programs; however, the strength of evidence linking these effects directly to human cancer prevention varies substantially across metabolites and tissues [15,16,44,45].

Among microbiota-derived metabolites, short-chain fatty acids (SCFAs)—particularly acetate, propionate and butyrate—are important products of microbial fermentation of dietary fiber and resistant starch [15,36]. Human colonic measurements indicate total SCFA concentrations of roughly 80–130 mmol/kg, commonly with an acetate:propionate:butyrate ratio near 60:20:20, whereas peripheral circulating concentrations are much lower [36]. Butyrate is a well-characterized HDAC inhibitor and can induce histone hyperacetylation, differentiation, and apoptosis in colorectal cancer models [12,17]. Recent metabolomic and chromatin-mapping studies further indicate that butyrate and propionate can modify histone acylation marks and transcriptional programs in a nutrient-dependent manner [35]. The large gradient between luminal and systemic exposure is important when evaluating whether experimental concentrations are physiologically attainable in specific tissues [35,36].

Beyond histone acetylation, microbiota-derived metabolites may influence DNA methylation and other epigenetic processes through one-carbon metabolism, metabolite availability, and signaling pathways that intersect with DNMT activity and chromatin regulation [44,45]. These microbiome–epigenome interactions can intersect with inflammatory, oxidative-stress, and cell-cycle pathways implicated in carcinogenesis, but causal evidence in humans remains incomplete [44,45].

Diet-induced dysbiosis, commonly associated with Western dietary patterns rich in saturated fats and refined carbohydrates, promotes chronic inflammation and aberrant epigenetic programming linked to tumor development [46]. In contrast, fiber-rich, polyphenol-rich, and omega-3–enriched dietary patterns support a eubiotic microbiota that favors anti-inflammatory and tumor-suppressive epigenetic signaling [1]. Importantly, microbiota-mediated epigenetic regulation is not confined to the colon but may exert systemic effects through immune modulation and metabolic signaling networks [18].

Collectively, experimental evidence suggests a mechanistic framework in which dietary inputs alter microbial composition and metabolism, while SCFAs and other microbial products can influence chromatin regulation. The strength of this evidence is greatest in cell and animal models. In humans, fecal or circulating SCFA concentrations are imperfect proxies for tissue exposure, and direct demonstration that diet-induced SCFA changes produce durable, cancer-preventive epigenetic effects remains limited. These uncertainties are central to the Nutrigenomics–Microbiome–Epigenome Axis and should temper causal interpretation.

Figure 1: Conceptual schematic illustrating the Nutrigenomics–Microbiome–Epigenome Axis in cancer prevention: Dietary components shape gut microbiota composition and metabolic activity, leading to the production of short-chain fatty acids (SCFAs). SCFAs modulate epigenetic regulators such as histone deacetylases (HDACs) and DNA methyltransferases (DNMTs), thereby influencing gene expression programs involved in tumor suppression and oncogene regulation. These interconnected pathways collectively contribute to modulation of cancer risk.

## 7. Dietary Patterns and Epigenomic Profiles

While individual nutrients exert measurable epigenetic effects, dietary patterns provide a more integrative view of long-term epigenetic modulation. Population-based studies increasingly associate specific dietary patterns with distinct epigenomic signatures relevant to cancer prevention.

### 7.1. Mediterranean Diet

While Figure 1 illustrates the mechanistic Nutrigenomics–Microbiome–Epigenome Axis at the molecular level, habitual dietary patterns represent complex, correlated exposures rather than isolated nutrients. Human intervention and epigenomic studies suggest that dietary-pattern adherence can be associated with changes in DNA methylation and other molecular endpoints, but results are heterogeneous and do not yet establish that such epigenetic changes mediate cancer-risk reduction [25,37,47].

In this review, the Mediterranean diet is defined as a predominantly plant-forward pattern characterized by high intake of vegetables, fruits, legumes, whole grains, nuts and seeds; olive oil as the principal added fat; regular fish/seafood intake; moderate dairy and poultry; and low intake of red and processed meat, refined products and sweets [5,31,37,38]. This description reflects the core components shared across commonly used Mediterranean-diet adherence scores, although individual studies differ in scoring thresholds, serving definitions, inclusion of alcohol, and whether adherence is assessed relative to cohort-specific medians or predefined targets. These methodological differences should be considered when comparing epigenetic associations across cohorts. Adherence to this dietary pattern has been associated with DNA methylation differences in genes involved in inflammation, oxidative stress, and tumor-related pathways [37,38].

Mediterranean-diet components can plausibly influence SCFA production, polyphenol exposure, lipid signaling and epigenetic regulation; human studies have reported diet-associated differences in DNA methylation and regulatory RNAs [37,38,47]. However, direct evidence that the Mediterranean diet produces sustained HDAC inhibition or a uniformly “balanced” DNMT state in humans is insufficient. Accordingly, these mechanisms should be considered biologically plausible contributors rather than established mediators of the lower cancer risk observed in epidemiological studies [31,37,38,47].

### 7.2. Western Diet

In this review, a Western dietary pattern refers to a food pattern characterized by high intake of red and processed meats, refined grains, ultra-processed foods, added sugars/sugar-sweetened beverages, saturated fat and sodium, together with comparatively low intake of vegetables, fruits, legumes, whole grains and dietary fiber [3,39,40,46]. It is not a single prescriptive diet; epidemiological studies generally derive Western-pattern scores empirically from food-frequency or dietary-record data, and the exact food loadings therefore vary by population. Such patterns are associated with microbiota dysbiosis, reduced saccharolytic fermentation and pro-inflammatory signaling; however, direct attribution of specific epigenetic changes to the overall pattern remains vulnerable to confounding by adiposity, smoking, physical activity and other correlated lifestyle factors [3,39,40,46].

Animal models further demonstrate that sustained exposure to Western dietary patterns induces persistent epigenetic alterations, some of which may extend across generations, highlighting the long-term biological imprint of dietary habits [40].

### 7.3. Caloric Restriction and Fasting-Mimicking Diets

Caloric restriction and fasting-mimicking interventions represent metabolic strategies that converge on epigenetic regulators central to the diet–epigenome axis. These interventions influence sirtuin signaling (SIRT1), AMP-activated protein kinase (AMPK), and mTOR pathways, thereby modulating chromatin structure, histone acetylation, and transcriptional stability [21,33,41].

By promoting epigenetic configurations associated with genomic stability and reduced proliferative signaling, caloric restriction paradigms provide additional support for the concept that sustained dietary patterns can reshape epigenomic landscapes relevant to cancer prevention.

## 8. Epigenetic Biomarkers and Precision Cancer Prevention

Epigenetic biomarkers offer promising tools for early cancer detection, risk stratification, and monitoring of dietary interventions. DNA methylation signatures in blood, saliva, and stool samples have demonstrated potential as non-invasive biomarkers for cancer risk assessment [6,48].

Nutrigenomic approaches integrating epigenetic biomarkers may enable personalized dietary recommendations tailored to individual epigenetic and genetic profiles [7]. However, validation of diet-responsive epigenetic markers in large, longitudinal human cohorts remains limited.

## 9. Evidence from Human Studies and Clinical Trials

Although mechanistic evidence supports diet-responsive epigenetic regulation, translation to human cancer prevention remains complex. Human intervention studies, observational cohorts, EWAS, and systematic reviews investigating methyl donors, dietary patterns, and other nutritional exposures report heterogeneous effects across tissues, populations, doses, and epigenetic endpoints [25,49,50].

Intervention studies examining folate and one-carbon metabolism nutrients have demonstrated measurable changes in global and gene-specific DNA methylation patterns, particularly in colorectal cancer risk contexts [13,14]. However, dose-dependent dual effects have been reported, whereby folate deficiency is associated with global hypomethylation and genomic instability, while excessive supplementation may promote the progression of pre-existing neoplastic lesions [14]. These findings highlight the importance of individualized risk stratification when targeting DNA methylation pathways.

Human studies of curcumin, resveratrol, and green tea catechins provide substantially more limited epigenetic evidence than preclinical experiments. Curcumin trials in colorectal neoplasia have mainly assessed pharmacokinetics and cancer-related biomarkers at gram-level supplemental doses (e.g., 2–4 g/day), rather than demonstrating direct DNMT inhibition or histone remodeling in vivo [32]. A phase I study of resveratrol-containing preparations in patients with colon cancer detected modulation of Wnt-pathway gene expression in normal colonic mucosa, but did not establish a generalized epigenetic mechanism [34]. Likewise, randomized green tea extract studies in men with prostate cancer have demonstrated tissue exposure to catechins; however, one study found no significant difference in methylation activity between intervention groups despite measurable EGCG in prostate tissue [26]. Thus, clinical data support target-tissue exposure and selected biomarker effects more strongly than they support direct, reproducible modulation of DNMT activity, histone acetylation, or circulating regulatory-RNA profiles. The broader 2024 systematic review of nutritional interventions and DNA methylation outcomes similarly reported heterogeneous effects across populations and interventions [25].

Evidence linking microbiota-derived SCFAs, particularly butyrate, to epigenetic regulation is strongest in experimental colorectal models [12,17,35]. Human studies support the relationship between fermentable-fiber intake, microbial SCFA production, and host metabolic responses, but direct evidence connecting these changes to reproducible cancer-relevant epigenetic signatures is limited [15,36,44]. Direct interventional demonstration of sustained HDAC inhibition in human target tissues remains particularly sparse. Beyond single-nutrient approaches, small human dietary/lifestyle interventions have reported changes in DNA methylation clocks, but these findings require replication and should not be interpreted as evidence of reduced cancer risk [25,51].

Population-based studies evaluating **Mediterranean dietary patterns** consistently associate adherence with favorable DNA methylation profiles in genes involved in inflammation, oxidative stress, and tumor suppression [31,37,38]. Conversely, Western dietary patterns correlate with pro-inflammatory epigenetic signatures, including global DNA hypomethylation and dysregulated miRNA expression linked to colorectal and breast cancer risk [3,39,40]. These findings suggest that whole dietary patterns may exert cumulative epigenetic effects beyond individual nutrient supplementation.

Emerging data also indicate that **caloric restriction and fasting-mimicking interventions** influence epigenetic regulators such as SIRT1 and the AMPK–mTOR axis, potentially promoting chromatin states associated with genomic stability and tumor suppression [21,33,41]. However, robust long-term randomized controlled trials with cancer incidence as an endpoint are still lacking. Emerging epigenome-wide association studies (EWAS) further support population-level links between dietary patterns and differential DNA methylation signatures across multiple genomic loci, strengthening the epidemiological basis for precision nutrition approaches in cancer prevention [49].

Overall, human studies indicate that dietary interventions can modify selected DNA methylation outcomes, but the evidence is heterogeneous. A 2024 systematic review of clinical trials identified variable effects across healthy dietary patterns, specific nutrients and supplements, including conflicting findings for folic acid and polyunsaturated fatty acids [25]. Nutritional EWAS add genome-wide resolution, yet many associations remain cross-sectional, tissue-specific, and vulnerable to residual confounding; replication across populations and ancestries is still limited [49]. Large EWAS meta-analyses demonstrate that dietary exposures can associate with numerous CpG sites, but association does not establish that these methylation differences mediate cancer risk [50].

Global methylation measures such as LINE-1 are attractive because they are technically accessible, but they provide limited locus specificity and may not reflect regulatory changes in the tissue of cancer origin. Epigenetic clocks offer a complementary systems-level measure of biological aging; small intervention studies have reported diet-associated changes in epigenetic age, but these findings require independent replication and should not be interpreted as evidence of reduced cancer incidence [25,51]. Likewise, circulating miRNAs and cell-free DNA methylation signatures are promising biomarkers, yet pre-analytical variability, cell-of-origin effects, assay standardization, and uncertain clinical thresholds remain substantial barriers. Taken together, current human evidence supports biological responsiveness of the epigenome to diet more strongly than it supports clinical utility for cancer prevention.

**Figure 3:**
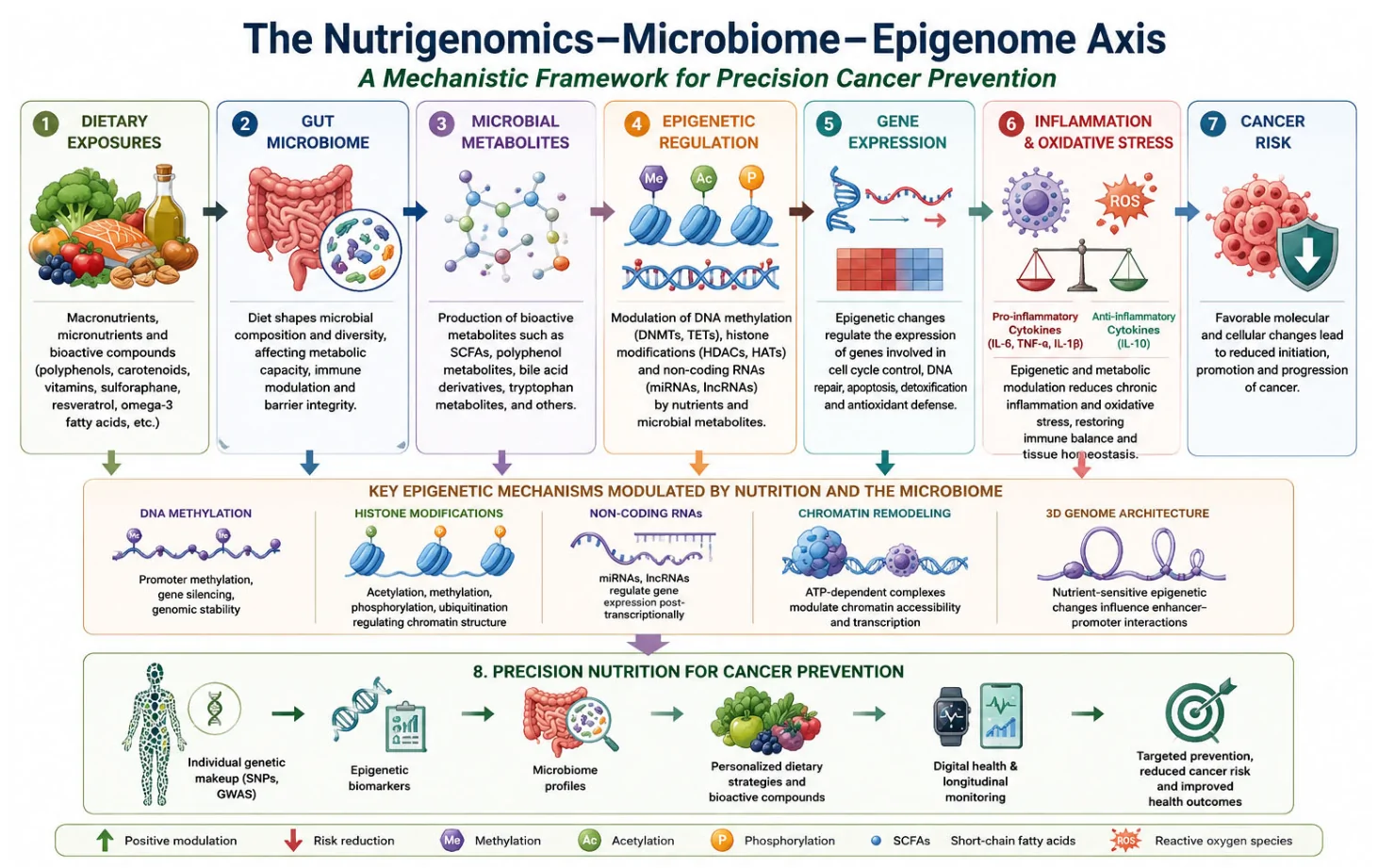
The Nutrigenomics–Microbiome–Epigenome Axis: A conceptual framework for precision cancer prevention.

Building on this critical appraisal, we use the Nutrigenomics–Microbiome–Epigenome Axis as a conceptual framework rather than as an established causal pathway. The proposed network integrates dietary exposures, microbial metabolism, epigenetic regulation and gene expression, consistent with evidence that these processes interact across metabolic, inflammatory and immune pathways [11,15,44,45]. Importantly, evidence strength differs across individual links in this network. Its principal value is therefore to identify testable interfaces for longitudinal, intervention, and multi-omics studies rather than to imply that a validated clinical algorithm is currently available (Figure 3).

Figure 3 proposed conceptual framework illustrating the biological interactions between dietary exposures, the gut microbiome, microbial metabolites, epigenetic regulation, and gene expression in cancer prevention. Dietary bioactive compounds modulate microbial composition and metabolic activity, generating signaling molecules that influence DNA methylation, histone modifications, chromatin remodeling, and non-coding RNA expression. These epigenetic mechanisms regulate inflammation, oxidative stress, and cancer-related pathways, ultimately contributing to precision nutrition strategies aimed at personalized cancer prevention and improved long-term health outcomes.

## 10. Challenges, Limitations, and Ethical Considerations

Despite compelling mechanistic evidence, translation into routine practice remains limited by several interacting barriers. Human diets are complex exposures that are difficult to quantify precisely, and epigenetic responses vary by tissue, cell composition, age, sex, ancestry, genetic background, medication use, and microbiome state [4,10]. Biomarker studies additionally face batch effects, platform differences, pre-analytical variability, and the absence of clinically validated reference ranges. These factors reduce reproducibility and complicate the definition of actionable thresholds for individual patients.

Another critical challenge concerns dose and timing. Nutrients such as folate exhibit dual effects, where deficiency increases cancer risk, while excessive supplementation may promote tumor progression in individuals with pre-existing lesions [14]. This underscores the importance of precision rather than generalized dietary recommendations.

From an ethical perspective, nutrigenomic and epigenetic profiling raises concerns related to data privacy, informed interpretation, health disparities, and equitable access [52]. Clinical implementation would also require analytical validation, demonstration of incremental benefit over conventional risk assessment, cost-effectiveness evidence, interoperable data infrastructure, professional training and clear regulatory standards. Until these requirements are met, nutrigenomic and epigenetic biomarkers should be regarded primarily as research tools rather than routine determinants of cancer-prevention advice.

## 11. Future Perspectives

The conceptual framework proposed in this review (Figure 2) illustrates the dynamic interactions between nutrition, the gut microbiome and epigenetic regulation, highlighting the importance of integrated biological approaches in future cancer prevention research. As multi-omics technologies, artificial intelligence, and systems biology continue to evolve, this framework may contribute to the development of predictive models capable of supporting precision nutrition and personalized cancer prevention strategies. The future of nutrigenomics in cancer prevention lies in the integration of multi-omics technologies, including genomics, epigenomics, transcriptomics, metabolomics, and microbiomics [11]. Such integrative approaches will enable a systems-level understanding of diet–epigenome interactions and their impact on carcinogenesis.

Artificial intelligence and machine learning tools are expected to play a central role in identifying epigenetic signatures predictive of cancer risk and dietary responsiveness [53]. Additionally, advances in liquid biopsy technologies may facilitate the use of epigenetic biomarkers for real-time monitoring of dietary interventions [54].

Importantly, early-life nutrition represents a critical window for epigenetic programming with long-term implications for cancer risk [24]. Future preventive strategies may therefore focus not only on adult dietary interventions but also on maternal and childhood nutrition as determinants of lifelong epigenetic health.

## 12. Conclusions

Current evidence indicates that dietary exposures can modify cancer-relevant epigenetic processes, including DNA methylation, histone regulation and non-coding RNA expression, directly and through microbiome-derived metabolites. However, human findings remain heterogeneous, tissue-specific, and insufficient to establish that these molecular changes causally reduce cancer incidence [25,49].

Accordingly, the Nutrigenomics–Microbiome–Epigenome Axis is best regarded as a hypothesis-generating framework rather than a validated clinical pathway. Precision nutrition for cancer prevention requires prospective and randomized validation of reproducible biomarkers, clinically relevant dietary exposures, and incremental benefit beyond established prevention strategies before routine implementation can be justified [7,25].

## Data Availability

No new datasets were generated or analyzed during the current study. This article is based exclusively on previously published literature, and all sources supporting the findings are appropriately cited within the manuscript.

## Abbreviations

The following abbreviations are used in this manuscript:

**Abbreviation**	**Definition**
AI	Artificial Intelligence
APC	Article Processing Charge
BMI	Body Mass Index
CRC	Colorectal Cancer
CRISPR	Clustered Regularly Interspaced Short Palindromic Repeats
DNA	Deoxyribonucleic Acid
DNMT	DNA Methyltransferase
EGCG	Epigallocatechin-3-Gallate
EMT	Epithelial–Mesenchymal Transition
ER	Estrogen Receptor
FA	Fatty Acid
FTO	Fat Mass and Obesity-Associated Gene
GPCR	G Protein-Coupled Receptor
HDAC	Histone Deacetylase
HAT	Histone Acetyltransferase
IL	Interleukin
KEGG	Kyoto Encyclopedia of Genes and Genomes
lncRNA	Long Non-Coding RNA
MAPK	Mitogen-Activated Protein Kinase
miRNA	MicroRNA
mRNA	Messenger RNA
mTOR	Mammalian Target of Rapamycin
NAD+	Nicotinamide Adenine Dinucleotide
NGS	Next-Generation Sequencing
NF-κB	Nuclear Factor Kappa B
Nrf2	Nuclear Factor Erythroid 2–Related Factor 2
NCDs	Non-Communicable Diseases
OMICS	High-Throughput Molecular Profiling Technologies
PI3K	Phosphoinositide 3-Kinase
PPAR	Peroxisome Proliferator-Activated Receptor
PR	Progesterone Receptor
RNA	Ribonucleic Acid
ROS	Reactive Oxygen Species
SCFA	Short-Chain Fatty Acid
SNP	Single Nucleotide Polymorphism
TCA Cycle	Tricarboxylic Acid Cycle
TET	Ten-Eleven Translocation
TGF-β	Transforming Growth Factor Beta
TNF-α	Tumor Necrosis Factor Alpha
VEGF	Vascular Endothelial Growth Factor
Wnt	Wingless/Integrated Signaling Pathway
